# Beyond Budget Execution: Financial and Operational Indicator Profiles in Bacău County’s Maternal and Child Health Programme, Romania, 2021–2024

**DOI:** 10.3390/healthcare14152259

**Published:** 2026-07-24

**Authors:** Oana Lia Botezatu (Mocondoi), Veronica Mocanu, Irina Mihaela Eșanu, Dana-Teodora Anton-Păduraru, Mariana Postolache, Elena Mihaela Cărăușu

**Affiliations:** 1 Doctoral School, “Grigore T. Popa” University of Medicine and Pharmacy, 700115 Iași, Romania; 2 Center for Obesity BioBehavioral Experimental Research, Department of Morpho-Functional Sciences II (Pathophysiology), Faculty of Medicine, “Grigore T. Popa” University of Medicine and Pharmacy, 700115 Iași, Romania; veronica.mocanu@umfiasi.ro; 3 Department of Medical Specialties I, Faculty of Medicine, “Grigore T. Popa” University of Medicine and Pharmacy, 700115 Iași, Romania; irina.esanu@umfiasi.ro; 4 Department of Mother and Child Medicine, Faculty of Medicine, “Grigore T. Popa” University of Medicine and Pharmacy, 700115 Iași, Romania; dana.anton@umfiasi.ro; 5 Discipline of Public Health, Department of Dental Medicine II, Faculty of Dental Medicine, “Carol Davila” University of Medicine and Pharmacy, 020021 Bucharest, Romania; 6 “Grigore T. Popa” University of Medicine and Pharmacy, 700115 Iași, Romania; elena.carausu@umfiasi.ro

**Keywords:** maternal and child health, programme evaluation, health system performance, budget execution, administrative data, Romania, public health management

## Abstract

**Highlights:**

**What are the main findings?**

- Similar budget execution rates across programme interventions were associated with substantial differences in activity volume, reported coverage, and mean cost per beneficiary.
- Neonatal hearing screening generated the highest activity volume, but reported coverage varied considerably across years.

**What are the implications of the main findings?**

- Budget execution alone is insufficient to assess public health programme performance and should be complemented by operational indicators.
- Multidimensional monitoring at the county level may support better resource allocation and earlier identification of implementation bottlenecks.

**Abstract:**

**Background/Objectives:** Financial monitoring of public health programmes frequently relies on budget execution indicators, which may not fully reflect operational performance. This study evaluated the financial and operational performance of Romania’s National Programme for Women’s and Children’s Health at the county level and examined how resource utilisation coexisted with programme activity, coverage of the eligible population, and local implementation profiles during 2021–2024. **Methods:** A longitudinal descriptive observational study was conducted using aggregated administrative data from Bacău County, Romania. Financial indicators included annual budget allocations, actual expenditures, budget execution rates, and mean cost per beneficiary. Operational indicators included activity volume, reported coverage of eligible populations, and continuity of implementation. Monetary values were converted into constant 2024 prices. Descriptive and comparative trend analyses were performed. **Results:** The mean budget execution rate was 84.08%, ranging from 68.70% to 94.93%. Screening interventions generated the highest activity volumes and lowest unit costs, whereas prophylactic and nutritional interventions accounted for most programme expenditure. Reported coverage varied substantially across interventions, particularly for neonatal hearing screening. Similar budget execution rates were associated with distinct operational profiles. **Conclusions:** Budget execution alone did not adequately reflect programme performance. Integrated monitoring of financial and operational indicators may support more effective programme management and resource allocation.

## 1. Introduction

Performance evaluation of public health programmes is a central component of health systems management because it examines how allocated resources are transformed into services and measurable outputs for eligible populations. Linking financial resources with programme activity is essential for understanding how efficiently programmes operate under real-world implementation conditions. When financial and operational indicators are assessed separately, important aspects of programme functioning may remain insufficiently captured [1].

Conceptual frameworks developed by the World Health Organization and the Organisation for Economic Co-operation and Development describe programme performance through relationships between inputs, processes, outputs, and outcomes, supporting managerial decision-making and efficient use of public resources [1,2]. These approaches are consistent with broader health system performance models that emphasise the relationship between structure, processes, and outcomes [3,4]. More recent frameworks additionally highlight resilience, equity, people-centredness, and sustainability as relevant dimensions of health system performance [5,6].

Within these models, efficiency refers to the relationship between resources used and services delivered or outputs achieved and should be distinguished from effectiveness, quality, and impact [1,2]. This distinction is particularly relevant in studies based on administrative data, where routinely collected records can support the evaluation of resource utilisation and service delivery but usually do not permit direct attribution of changes in health outcomes. Administrative datasets are increasingly recognised as useful sources for programme monitoring and implementation assessment, especially when primary data collection is costly or impractical [7,8].

Despite the growing emphasis on integrated performance measurement, evaluations of public health programmes are often conducted separately for financial and operational dimensions. Budget execution provides information on the use of allocated funds but does not directly reflect service utilisation, continuity of implementation, or population coverage. Operational indicators describe activity volume and intermediate outputs, but their interpretation is incomplete when disconnected from the resources consumed. This fragmentation has been identified as an important limitation of performance assessment and may reduce the usefulness of routine reporting for evidence-informed management [9,10,11].

This issue is particularly relevant for maternal and child health programmes, which commonly involve multiple interventions, diverse beneficiary groups, and coordination across primary care, hospital services, and public health authorities. International evidence indicates that programme performance in maternal and child health is strongly influenced by organisational capacity, continuity of care pathways, quality of service delivery, and local implementation context [12,13,14]. Consequently, similar levels of expenditure may generate different operational outputs across settings. Published subnational evidence examining the relationship between financial inputs and operational programme performance remains relatively limited in Central and Eastern Europe. Following the post-communist transition period, countries in the region have implemented extensive reforms affecting health financing, governance arrangements, and service delivery structures. These changes have increased the importance of evaluating how national programmes are implemented and managed at local level, particularly in health systems characterised by heterogeneous organisational contexts [15,16]. Health reforms in the region have frequently focused on financing mechanisms and governance arrangements, while less attention has been given to routine county- or district-level implementation performance [15,16]. Romania represents a relevant case because several major public health programmes are nationally financed but locally implemented through heterogeneous institutional arrangements.

Romania’s National Programme for Women’s and Children’s Health includes preventive, screening, nutritional, and therapeutic interventions delivered through multiple providers with distinct reporting responsibilities. In such a system, budget execution does not automatically indicate service reach or implementation performance, while operational indicators cannot be adequately interpreted without reference to the financial resources used. Existing evaluations are conducted predominantly at the national level using aggregated indicators, which may obscure local variation in administrative capacity, service organisation, and programme delivery [10,15]. County-level analyses may therefore complement national reporting by identifying implementation heterogeneity and managerial bottlenecks insufficiently visible in centralised datasets [17,18,19].

The county analysed in this study provides a relevant setting because it has a mixed urban–rural population structure, a public healthcare delivery network typical of a medium-sized Romanian county, and complete administrative reporting for the study period. In this context, the present study aimed to conduct an integrated evaluation of the financial and operational performance of Romania’s National Programme for Women’s and Children’s Health at the county level during 2021–2024 using routinely collected administrative data. Specifically, the study examined how resource utilisation was associated with programme activity, coverage of eligible populations, and local implementation profiles across programme interventions. The study contributes to the limited subnational evidence available on maternal and child health programme performance in Central and Eastern Europe. Its methodological contribution lies in the integrated assessment of financial and operational indicators using routinely collected administrative data, allowing programme implementation to be examined from multiple complementary perspectives.

## 2. Materials and Methods

### 2.1. Study Design and Setting

A retrospective longitudinal observational study with a county-level administrative case study design was conducted using routinely collected aggregated data for the period 2021–2024. The analysis covered four consecutive annual reporting cycles within Romania’s National Programme for Women’s and Children’s Health. This design was considered appropriate for evaluating programme implementation under real-world operational conditions using secondary administrative data. The study followed general reporting principles for observational studies, including those reflected in the STROBE framework [13].

The study was performed in one Romanian county selected as an analytically relevant setting because of its mixed urban–rural population structure, public healthcare delivery network typical of a medium-sized county, and complete reporting availability for the study period. The county had a resident population of approximately 600,000 inhabitants during the study period and recorded between 4148 and 5961 births annually. Programme implementation involved public maternity hospitals, primary care services, and healthcare providers participating in programme reporting and delivery. These characteristics support the analytical relevance of the selected county as a medium-sized Romanian administrative setting with complete programme reporting for the study period. A county-level approach was chosen because subnational analyses may identify implementation heterogeneity insufficiently visible in aggregated national reporting [10,15].

### 2.2. Data Sources and Data Collection

The evaluation used routinely collected administrative data generated through programme monitoring and financial reporting mechanisms. Financial data included annual approved budget allocations and recorded expenditures for each intervention component. Operational data were derived from annual reports submitted by healthcare providers involved in programme implementation and subsequently centralised by the county public health authority.

The final dataset consisted of aggregated annual intervention-level records covering the period 2021–2024. Variables referred to programme activity, beneficiary volume, and reported coverage of eligible populations. Before analysis, data were reviewed for completeness, internal consistency, and comparability across years. Validation procedures included verification of the concordance between reported numerators and denominators, assessment of annual reporting continuity, identification of missing values, and examination of logical consistency between operational and financial records. No independent external validation of source records was performed [7,8].

### 2.3. Variables and Indicators

Two categories of variables were analysed: financial and operational.

Financial indicators included annual budget allocations, actual expenditures, budget execution rate (actual expenditures/approved allocation × 100), and mean cost per beneficiary. To improve comparability over time, monetary values were converted into constant 2024 prices using an adjustment based on the national Consumer Price Index, consistent with standard approaches in health economics and public finance analyses [16].

Operational indicators included activity volume (number of beneficiaries or reported services, depending on the intervention), reported coverage of eligible populations, and continuity of implementation across years. Eligible populations were defined according to programme-specific administrative criteria. For neonatal hearing screening, the denominator consisted of all live births reported by participating maternity units. For retinopathy of prematurity screening, the denominator included newborns meeting programme eligibility criteria recorded within the reporting system. For prophylaxis of malnutrition in low birth weight infants and prophylaxis of Rh isoimmunisation syndrome, denominators were derived from programme-specific administrative records of eligible beneficiaries. For prophylaxis of dystrophy through provision of infant formula, the denominator consisted of the total population of children aged 0–12 months reported within the county monitoring system. Where clinically eligible populations were not directly available, proxy administrative denominators routinely used within the reporting system were applied. Coverage indicators derived from different denominators were therefore interpreted cautiously and were not considered directly comparable across all interventions [7,8]. Coverage indicators were constructed using different types of administrative denominators across interventions. For neonatal hearing screening, the denominator reflected all live births reported by participating maternity units. For ROP screening, malnutrition prophylaxis, and Rh isoimmunisation prophylaxis, the denominators reflected registered programme-specific eligible groups. For infant formula provision, the denominator reflected the total population of children aged 0–12 months, because a complete clinically eligible denominator was not available in the administrative dataset. Therefore, coverage indicators were used to describe intervention-specific implementation patterns and were not used to rank interventions or infer relative effectiveness.

### 2.4. Analytical Framework

The analytical strategy was descriptive and exploratory and aimed to assess programme implementation patterns rather than programme effectiveness, clinical outcomes, or causal relationships. Annual trends in allocations, expenditures, budget execution, activity volume, reported coverage, and mean costs were examined comparatively across interventions and over time.

An integrated assessment was conducted by jointly examining financial indicators (budget execution and mean cost) together with operational indicators (activity volume, reported coverage, and continuity of implementation). No predefined thresholds, scoring systems, classification matrices, or performance categories were applied. The integrated financial–operational profile was derived through descriptive comparison of financial and operational indicators across programme interventions and over time, with the purpose of identifying implementation patterns rather than ranking interventions according to performance. This multidimensional approach is consistent with contemporary health system performance frameworks that recommend linking resources, processes, and outputs in routine service evaluation [6,10].

Continuity of implementation was defined as the presence of reported activity for a given intervention in each year of the study period. In addition to annual activity reporting, continuity was assessed descriptively by examining the relative stability of activity volume and reported coverage across consecutive years. No predefined thresholds or quantitative criteria were applied. The indicator was used to characterise implementation patterns and programme continuity over time rather than as a formal measure of performance.

### 2.5. Statistical Analysis

Statistical analysis was descriptive. Continuous indicators were summarised using means, standard deviations, ranges, and coefficients of variation, where appropriate [17,18]. These measures were used to describe annual variability and relative stability across programme components.

Inferential statistical testing and correlation modelling were not performed because the study was based on four annual aggregated observations per indicator and had an exploratory administrative-data design, making formal inferential interpretation inappropriate [18]. Instead, relationships between expenditures and reported activity were assessed through comparative examination of annual trends and cross-intervention profiles.

Data processing and statistical calculations were performed using Microsoft Excel 365 (Microsoft Corporation, Redmond, WA, USA) and IBM SPSS Statistics, version 28 (IBM Corp., Armonk, NY, USA).

### 2.6. Ethical Considerations

The study used exclusively aggregated administrative data without personal identifiers. No individual-level health records were accessed. The analysis was conducted within a doctoral research project approved by the Research Ethics Committee of “Grigore T. Popa” University of Medicine and Pharmacy, Iași, Romania (Registration No. 255/17 January 2023), under Doctoral Contract No. 1916/29 October 2021. The study complied with applicable principles governing the secondary use of anonymised administrative data in health research [13]. The administrative records for 2021–2022 had been generated through routine programme reporting before ethical approval. Their secondary use for research purposes, including the compilation and analysis of the study dataset, was conducted after ethical approval was obtained on 17 January 2023.

Declaration on the Use of Generative Artificial Intelligence: Generative artificial intelligence tools were used to assist with language editing and improving the clarity of the manuscript. The authors reviewed and approved the final content and take full responsibility for the article.

## 3. Results

### 3.1. Financial Performance

**Figure 1:**
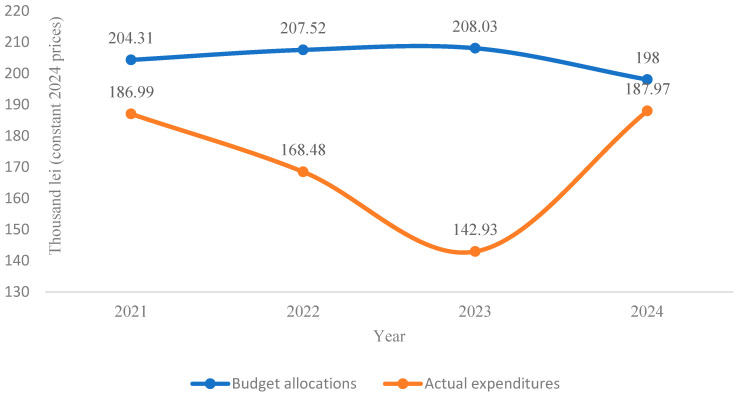
Annual budget allocations and actual expenditures of the programme during 2021–2024 (thousand lei, constant 2024 prices).

After inflation adjustment to constant 2024 prices, total programme budget allocations remained broadly stable during 2021–2023 (204.31–208.03 thousand lei), followed by a modest decline to 198.00 thousand lei in 2024 (Table 1; Figure 1).

Actual expenditures fluctuated more than allocations, decreasing from 186.99 thousand lei in 2021 to 142.93 thousand lei in 2023, before recovering to 187.97 thousand lei in 2024. The gap between approved allocations and actual expenditures was widest in 2023 and narrowed substantially in 2024 (Figure 1).

The aggregate budget execution rate varied between 68.70% and 94.93% across the study period. Following a decline from 2021 to 2023, the rate increased markedly in 2024. Across 2021–2024, the mean execution rate was 84.08%.

The distribution of budget allocations differed across intervention components. Screening interventions received smaller allocations, whereas prophylactic and nutritional interventions accounted for the largest share of programme funding (Supplementary Table S1).

At intervention level, annual variability in budget execution was more pronounced for screening components than for prophylactic and nutritional interventions (Supplementary Table S2).

### 3.2. Operational Performance

Operational indicators revealed marked heterogeneity across programme interventions in activity volume, reported coverage, and implementation stability.

Activity volume was concentrated in screening interventions. Neonatal hearing screening accounted for 76.32% of the total aggregated programme activity volume during 2021–2024. Prophylaxis of malnutrition and prophylaxis of Rh isoimmunisation syndrome accounted for 9.18% and 6.55% of total activity, respectively. The remaining interventions had smaller shares (Table 2).

Reported coverage of eligible populations differed substantially across interventions (Table 3).

Retinopathy of prematurity screening recorded the highest mean reported coverage (96.51%), including administratively reported values of 100% during 2022–2024. Prophylaxis of malnutrition showed a high mean coverage (90.73%) with a progressive increase over time. Prophylaxis of Rh isoimmunisation syndrome had a mean coverage of 70.86%.

Neonatal hearing screening showed the greatest annual variation in reported coverage, ranging from 45.03% to 74.61%. By contrast, coverage for prophylaxis of dystrophy through provision of infant formula remained low throughout the study period (mean 3.21%). As this indicator was calculated using the total population of children aged 0–12 months rather than only clinically eligible beneficiaries, direct comparison with other interventions should be interpreted cautiously (Table 3).

Implementation continuity differed across programme components. Annual series of reported activity volume and coverage suggested greater stability for interventions with consistently high reported coverage, whereas other components showed more variable operational patterns.

Overall, screening interventions generated the largest share of programme activity, while prophylactic and therapeutic interventions had lower volumes and distinct coverage profiles.

### 3.3. Integrated Financial–Operational Profile

Descriptive comparison of financial and operational indicators revealed distinct financial–operational profiles across programme interventions, despite broadly similar mean budget execution rates (79.29–86.22%) (Table 4).

Mean cost per beneficiary varied substantially between interventions. Screening components had the lowest unit costs, ranging from 3.79 lei for neonatal hearing screening to 40.48 lei for retinopathy of prematurity screening. By contrast, lower-volume prophylactic and therapeutic interventions showed markedly higher mean costs, ranging from 207.43 lei to 241.00 lei per beneficiary.

Cost variability also differed across programme components. Prophylaxis of dystrophy through provision of infant formula and prophylaxis of Rh isoimmunisation syndrome showed the most stable cost profiles, whereas neonatal hearing screening and prophylaxis of malnutrition exhibited greater year-to-year fluctuations (Supplementary Table S3).

When financial and operational indicators were considered jointly, screening interventions were characterised by high activity volumes and low unit costs, whereas interventions targeting smaller beneficiary groups combined lower activity volumes with higher average costs. These descriptive differences should not be interpreted as evidence of greater efficiency because programme interventions differed in terms of clinical objectives, target populations, eligibility criteria, and implementation requirements.

Overall, similar levels of budget execution did not translate into comparable operational profiles, suggesting meaningful differences in implementation models and resource utilisation across programme interventions.

## 4. Discussion

The present study shows that relatively similar levels of budget execution can coexist with meaningful differences in operational performance across interventions included in the National Programme for Women’s and Children’s Health. Although financial resource use was broadly comparable across the analysed components, clear variation was observed in activity volume, reported coverage, continuity of implementation, and cost stability. In particular, similar budget execution rates were associated with different output and cost profiles across programme interventions. These findings suggest that budget execution, when considered alone, does not adequately describe how public health programmes function and should be interpreted together with operational indicators [19,20].

A notable contribution of this study is the integrated financial and operational evaluation conducted at county level using administrative data collected over a four-year period. The international literature indicates that local and regional analyses can identify implementation differences that may not be fully visible in aggregated national reporting and can generate useful evidence for service management, particularly when supported by implementation-oriented approaches and adequate information systems [21,22]. Persistent vulnerabilities affecting child and adolescent health and wellbeing internationally further reinforce the value of locally applied performance assessment [23]. Such multidimensional evaluations appear less frequently reported for maternal and child health programmes in Central and Eastern Europe.

The findings suggest that interventions embedded within routine clinical pathways tended to show more favourable operational profiles, reflected in higher activity volumes and stronger reported coverage. Neonatal hearing screening generated the largest share of programme activity, while screening for retinopathy of prematurity achieved the highest reported coverage levels. However, neonatal hearing screening also showed substantial year-to-year variation in coverage, suggesting that high service volume alone does not necessarily ensure stable programme reach. Several factors, including differences in maternity unit participation, referral pathways, staffing continuity, reporting practices, or temporary organisational constraints, may plausibly contribute to the observed variation. However, these factors were not directly assessed and should be interpreted as potential explanatory hypotheses. These observations are consistent with the literature indicating that interventions incorporated into standardised care processes often achieve higher uptake and more stable implementation because they depend less on additional initiatives by beneficiaries or providers [24,25].

By contrast, interventions requiring active identification of beneficiaries, repeated visits, post-discharge follow-up, supply continuity, or coordination across multiple levels of care showed greater annual variability. International evidence identifies transitions between services and continuity of follow-up as vulnerable points in maternal and child health programmes, particularly where tracking mechanisms and information infrastructure are insufficiently developed [24,26,27]. The present findings are consistent with this interpretation and suggest that organisational complexity may influence implementation stability.

The integrated financial–operational analysis also suggests that programme performance depends not only on the amount of resources allocated, but on how effectively those resources are converted into services for eligible populations. Screening interventions had markedly lower mean costs per beneficiary and higher activity volumes, whereas interventions targeting smaller beneficiary groups showed lower volumes and higher average costs. This pattern is consistent with the health economics literature showing that services with broader coverage and standardised delivery pathways may benefit from economies of scale, while interventions aimed at smaller populations or requiring more complex logistics often involve higher average costs [16,19]. However, these differences should not be interpreted as evidence of inefficiency, since higher-cost interventions may reasonably reflect greater clinical complexity, specialised inputs, or narrower eligibility criteria.

An important methodological implication is that similar budget execution rates may conceal substantially different operational realities. Interventions with comparable expenditure absorption displayed contrasting levels of coverage, activity volume, and cost variability. Financial execution alone is therefore an incomplete proxy for programme success and may obscure underperformance or implementation bottlenecks if used as the principal monitoring metric [20]. For routine management, combining expenditure indicators with coverage, continuity, and unit-cost measures would provide a more informative basis for decision-making.

A key strength of the study lies in the simultaneous use of indicators for budget execution, mean cost per beneficiary, activity volume, reported coverage, continuity, and variability, allowing a more comprehensive assessment of programme performance than analyses based on a single dimension. Multidimensional approaches are increasingly recommended in the literature on health system performance assessment [10,28]. The use of real-world administrative data additionally provides a practical picture of how the programme functions under routine operational conditions, which is directly relevant for public health service management [14,15].

The findings also have direct managerial implications. Monitoring systems for public health programmes should move beyond reporting budget execution and systematically include indicators of reported coverage, continuity, mean cost per beneficiary, and operational variability. At the local level, interventions with greater fluctuations in activity may benefit from dedicated scheduling systems, active beneficiary follow-up, stronger interinstitutional coordination, and periodic audits of reporting quality. A differentiated management approach, adapted to the operational profile of each intervention, may be more effective than applying identical administrative mechanisms across all programme components [21,24,29].

Several limitations should be acknowledged. First, the analysis was based on annually aggregated administrative data, which limited assessment of intra-annual variation, service timeliness, and individual determinants of utilisation. Although data completeness, reporting continuity, and internal consistency were reviewed prior to analysis, the study relied on routinely collected administrative data and did not include independent validation of provider-level source records. Second, some eligible populations were estimated using proxy administrative denominators, and coverage indicators should therefore be interpreted cautiously. Third, the study period included only four annual observations, limiting the assessment of longer-term trends. Fourth, the case-study design based on one county restricts external generalisability, although analytical transferability to similar administrative settings remains plausible. Finally, the study did not assess clinical outcomes, patient experience, equity impacts, or cost-effectiveness, and no causal inferences can be drawn.

Overall, the study highlights the value of integrated evaluation of financial and operational indicators at subnational level. Similar levels of budget execution did not ensure comparable operational performance across interventions. Multidimensional monitoring approaches may support more precise managerial decisions, better prioritisation of vulnerable programme components, improved resource allocation, and stronger evidence-informed implementation of maternal and child health programmes. These conclusions are consistent with recent international recommendations emphasising the use of integrated performance assessment frameworks and stronger health information systems to support evidence-informed decision-making and health system improvement [5,22].

## 5. Conclusions

This study showed that relatively similar levels of budget execution across interventions included in the National Programme for Women’s and Children’s Health coexisted with substantial differences in operational performance. Marked variation was identified in activity volume, reported coverage, continuity of implementation, mean cost per beneficiary, and cost stability, indicating that financial execution alone does not provide a sufficient measure of programme performance.

The findings suggest that integrated monitoring frameworks combining financial and operational indicators can offer a more accurate understanding of how public health programmes function in practice. High-volume screening interventions were characterised by lower unit costs, whereas interventions targeting smaller beneficiary groups showed higher average costs and distinct implementation patterns. These differences should be interpreted in the context of variations in programme objectives, target populations, eligibility criteria, and operational complexity and should not be considered direct measures of relative efficiency. In addition, some components with high activity levels displayed variable coverage over time, highlighting that service volume alone does not necessarily reflect stable programme reach.

From a managerial perspective, routine monitoring should move beyond budget absorption and systematically include indicators of coverage, continuity, unit costs, and variability. Such an approach may support earlier identification of implementation bottlenecks, more appropriate allocation of resources, and better adaptation of management strategies to the specific profile of each intervention.

Overall, subnational multidimensional evaluations based on routinely collected administrative data may represent a practical tool for strengthening evidence-informed governance of maternal and child health programmes in Romania and in comparable health system settings.

## Figures and Tables

**Table 1:** Aggregate budget allocations, actual expenditures, and budget execution rate of the programme, 2021–2024 (thousand lei, constant 2024 prices).

Year	Budget Allocation	Actual Expenditures	Budget Execution Rate (%)
2021	204.31	186.99	91.52
2022	207.52	168.48	81.19
2023	208.03	142.93	68.71
2024	198.00	187.97	94.93

Note: Administrative data reported by programme providers; authors’ calculations based on nominal annual financial reports. Monetary values were inflation-adjusted and expressed in constant 2024 prices using the national Consumer Price Index.

**Table 2:** Distribution of reported programme activity volume (beneficiaries/services), 2021–2024.

No	Intervention	Cumulative Activity Volume (2021–2024)	Share of Total Activity (%)
1	Prevention of hearing impairment through neonatal hearing screening	10,293	76.32
2	Prevention of retinopathy of prematurity and its complications through neonatal screening, laser therapy, and disease follow-up monitoring	396	2.94
3	Prophylaxis of dystrophy in children aged 0–12 months who do not receive breast milk through provision of infant formula	675	5.01
4	Prophylaxis of malnutrition in children with low birth weight	1238	9.18
5	Prophylaxis of Rh isoimmunisation syndrome	884	6.55

Note: Shares were calculated by relating the cumulative volume of each intervention to the total aggregated programme activity volume for the period 2021–2024.

**Table 3:** Coverage indicators of eligible populations by intervention, 2021–2024.

No	Intervention	2021 (%)	2022 (%)	2023 (%)	2024 (%)	Mean (%)	Standard Deviation (%)
1	Prevention of hearing impairment through neonatal hearing screening	45.03	53.83	49.62	74.61	55.77	13.06
2	Prevention of retinopathy of prematurity and its complications through neonatal screening, laser therapy, and disease follow-up monitoring	86.05	100.00	100.00	100.00	96.51	6.98
3	Prophylaxis of dystrophy in children aged 0–12 months who do not receive breast milk through provision of infant formula	4.38	2.79	3.08	2.60	3.21	0.80
4	Prophylaxis of malnutrition in children with low birth weight	83.92	88.59	94.53	95.89	90.73	5.54
5	Prophylaxis of Rh isoimmunisation syndrome	68.34	69.23	75.40	70.45	70.86	3.17

Note 1: Indicators express the proportion of beneficiaries within the eligible population for each intervention during 2021–2024. Note 2: For prophylaxis of dystrophy through provision of infant formula, the indicator is calculated against the total population of children aged 0–12 months rather than only the clinically eligible population. Note 3: Reported values of 100% coverage reflect administrative data recorded within the programme monitoring system and should be interpreted as full reported coverage of the registered eligible population, not necessarily complete population-level coverage.

**Table 4:** Integrated financial and operational profile of programme interventions, 2021–2024.

No	Intervention	Mean Budget Execution Rate (%)	Mean Cost per Beneficiary (Lei, Constant 2024 Prices)	Cost Coefficient of Variation (%)
1	Prevention of hearing impairment through neonatal hearing screening	79.29	3.79	24.85
2	Prevention of retinopathy of prematurity and its complications through neonatal screening, laser therapy, and disease follow-up monitoring	85.27	40.48	15.67
3	Prophylaxis of dystrophy in children aged 0–12 months who do not receive breast milk through provision of infant formula	83.03	207.43	4.88
4	Prophylaxis of malnutrition in children with low birth weight	86.22	208.78	24.64
5	Prophylaxis of Rh isoimmunisation syndrome	85.14	241.00	6.70

Note: Mean costs were recalculated after inflation adjustment and are expressed in constant 2024 prices using the national Consumer Price Index. Budget execution rates and coefficients of variation remain unchanged because they are ratio-based indicators derived within the same annual period. The base year was 2024. Adjustment factors are shown in Supplementary Table S4.

## Data Availability

The data presented in this study are available from the corresponding authors upon reasonable request, subject to institutional and data protection regulations.

## Supplementary Materials

The following supporting information can be downloaded at: https://www.mdpi.com/article/10.3390/healthcare14152259/s1. Table S1: Trends in budget allocations and expenditures by intervention, 2021–2024 (thousand lei, constant 2024 prices); Table S2: Variability of the budget execution rate by intervention of the National Programme for Women’s and Children’s Health, 2021–2024; Table S3: Variability of mean operational costs across programme interventions, 2021–2024; Table S4: CPI adjustment procedure for financial indicators, 2021–2024.

## Abbreviations

The following abbreviations are used in this manuscript:

CPI	Consumer Price Index
OECD	Organisation for Economic Co-operation and Development
ROP	Retinopathy of Prematurity
SD	Standard Deviation
STROBE	Strengthening the Reporting of Observational Studies in Epidemiology
UNICEF	United Nations Children’s Fund
WHO	World Health Organization
