# Supplementary material for: Beyond Budget Execution: Financial and Operational Indicator Profiles in Bacău County’s Maternal and Child Health Programme, Romania, 2021–2024
Source: Healthcare (Basel). 2026 Jul 24;14(15):2259. doi: 10.3390/healthcare14152259 (PMC13464658; doi:10.3390/healthcare14152259)
Supplement: Supplementary file 1 [file healthcare-14-02259-s001.zip › healthcare-4312587-supplementary.pdf]

**Supplementary Table S1.** Trends in budget allocations and expenditures by intervention, 2021–2024 (thousand lei, constant 2024 prices)

| No | Intervention | Budget allocation | | | | Expenditures | | | |
|---|---|---|---|---|---|---|---|---|---|
| | | **2021** | **2022** | **2023** | **2024** | **2021** | **2022** | **2023** | **2024** |
| 1 | **Prevention of hearing impairment through neonatal hearing screening** | 9.29 | 8.16 | 26.40 | 22.00 | 9.00 | 6.80 | 9.83 | 21.95 |
| 2 | **Prevention of retinopathy of prematurity and its complications through neonatal screening, laser therapy, and disease follow-up monitoring** | 9.29 | 8.16 | 12.67 | 11.00 | 9.29 | 8.13 | 5.45 | 10.83 |
| 3 | **Prophylaxis of dystrophy in children aged 0–12 months who do not receive breast milk through the provision of infant formula** | 62.37 | 54.80 | 26.40 | 28.00 | 62.25 | 27.19 | 21.87 | 27.96 |
| 4 | **Prophylaxis of malnutrition in children with low birth weight** | 58.39 | 51.29 | 82.37 | 75.00 | 58.04 | 45.70 | 57.58 | 64.84 |
| 5 | **Prophylaxis of Rh isoimmunisation syndrome** | 65.03 | 85.12 | 60.19 | 62.00 | 48.45 | 72.53 | 49.26 | 61.39 |

*Notes: (1)* Administrative data reported by units involved in programme implementation; authors' calculations. (2) Monetary values were adjusted for inflation and are expressed in thousands of lei, constant 2024 prices, rounded to two decimal places. (3) Expenditures represent cumulative spending recorded by the end of each budget year.

**Supplementary Table S2.** Variability of the budget execution rate by intervention of the National Programme for Women's and Children's Health, 2021–2024

| No | Intervention | Mean budget execution rate (%) | Standard deviation (%) | Coefficient of variation (%) |
|---|---|---|---|---|
| 1 | **Prevention of hearing impairment through neonatal hearing screening** | 79.29 | 28.90 | 36.5 |
| 2 | **Prevention of retinopathy of prematurity and its complications** | 85.27 | 28.20 | 33.1 |
| 3 | **Prophylaxis of dystrophy in children aged 0–12 months who do not receive breast milk** | 83.03 | 23.70 | 28.6 |
| 4 | **Prophylaxis of malnutrition in children with low birth weight** | 86.22 | 12.20 | 14.1 |
| 5 | **Prophylaxis of Rh isoimmunisation syndrome** | 85.14 | 10.30 | 12.1 |

*Note:* Indicators were calculated using annual budget execution rates for each intervention during 2021–2024 (n=4), using descriptive statistics. Inflation adjustment was not required because budget execution rates are ratio measures calculated within the same year.

**Supplementary Table S3**. Variability of mean operational costs across programme interventions, 2021–2024

| No | Intervention | Mean cost (lei, constant 2024 prices) | Standard deviation (lei) | Coefficient of variation (%) |
|---|---|---|---|---|
| 1 | Prevention of hearing impairment through neonatal hearing screening | 3.79 | 0.94 | 24.85 |
| 2 | Prevention of retinopathy of prematurity and its complications through neonatal screening, laser therapy, and disease follow-up monitoring | 40.48 | 6.34 | 15.67 |
| 3 | Prophylaxis of dystrophy in children aged 0–12 months who do not receive breast milk through the provision of infant formula | 207.43 | 10.12 | 4.88 |
| 4 | Prophylaxis of malnutrition in children with low birth weight | 208.78 | 51.44 | 24.64 |
| 5 | Prophylaxis of Rh isoimmunisation syndrome | 241.00 | 16.15 | 6.70 |

*Note:* Indicators were calculated using annual mean cost values for 2021–2024 after inflation adjustment and are expressed in constant 2024 prices. The coefficient of variation was calculated as the percentage ratio between the standard deviation and the period mean.

**Table S4: CPI adjustment procedure for financial indicators, 2021–2024.**

| Year | CPI | Inflation rate | Adjustment factor to 2024 prices | Nominal allocation | Nominal expenditures | Allocation in constant 2024 prices | Expenditures in constant 2024 prices |
|---|---|---|---|---|---|---|---|
| 2021 | 105.05 | 5.05% | 1.3266 | 154.00 | 140.90 | 204.29 | 186.92 |
| 2022 | 113.80 | 13.80% | 1.1657 | 178.00 | 144.50 | 207.50 | 168.45 |
| 2023 | 110.40 | 10.40% | 1.0559 | 197.00 | 135.40 | 208.01 | 142.97 |
| 2024 | 105.59 | 5.59% | 1.0000 | 198.00 | 187.97 | 198.00 | 187.97 |
